# Supplementary material for: Study of Tribological Properties of Fullerenol and Nanodiamonds as Additives in Water-Based Lubricants for Amorphous Carbon (a-C) Coatings
Source: Nanomaterials (Basel). 2021 Dec 31;12(1):139. doi: 10.3390/nano12010139 (PMC8746735; doi:10.3390/nano12010139)
Supplement: Supplementary file 1 [file nanomaterials-12-00139-s001.zip › nanomaterials-1493694-supplementary.pdf]

nanomaterials

Supplementary Material

Study of Tribological Properties of Fullerenol and Nanodiamonds as Additives in Water-Based Lubricants for Amorphous Carbon (a-C) Coatings

Shuqing Chen [1,*], Qi Ding [2], Yan Gu [1], Xin Quan [1], Ying Ma [1], Yulong Jia [1], Hongmei Xie [1] and Jinzhu Tang [1,*]

1 College of Materials Science and Engineering, Yangtze Normal University, Chongqing 408100, China; 20170054@yznu.edu.cn (Y.G.); quanxinjianshe@163.com (X.Q.); yma2017@126.com (Y.M.); yljia2017@126.com (Y.J.); xiehongmei@yznu.cn (H.X.)
2 State Key Laboratory of Solid Lubrication, Lanzhou Institute of Chemical Physics, Chinese Academy of Sciences, Lanzhou 730000, China; dingqi@licp.cas.cn
* Correspondence: chenshuqing2021@163.com (S.C.); tangjinzhu11@163.com (J.T.); Tel.: +86-15023512606 (S.C.); +86-18875480116 (J.T.)

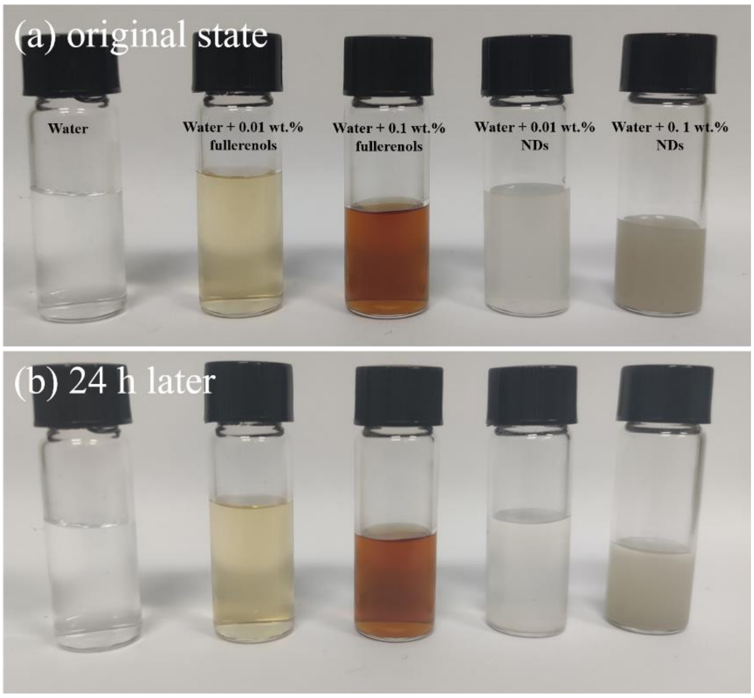

Figure S1. (**a**,**b**)Digital images of dispersion of fullerenol and nanodiamonds in water with various concentrations (c, wt%). The time for each picture is noted on the respective picture.

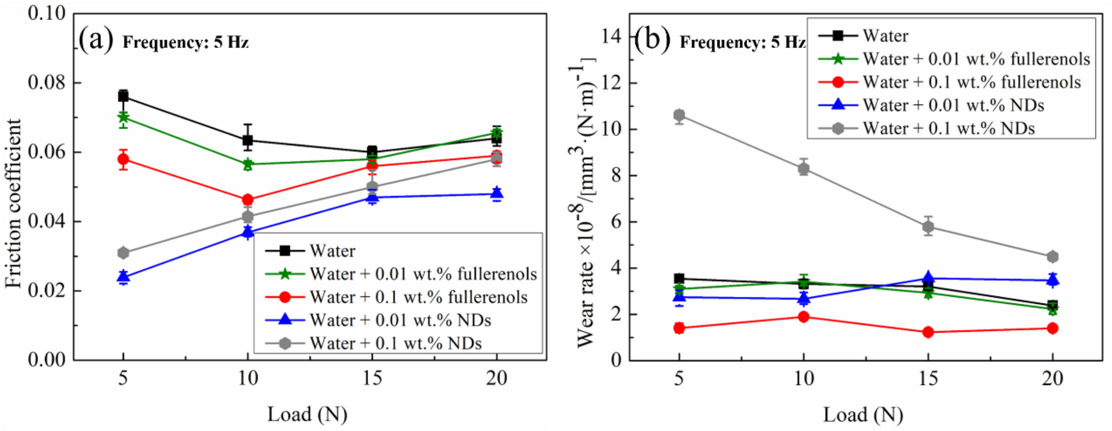

Figure S2. (**a**) Variation of COF with normal load and (**b**) variation of wear rate with normal load.
